# Functionalization of Phosphate and Tellurite Glasses and Spherical Whispering Gallery Mode Microresonators

**DOI:** 10.1021/acsomega.3c07075

**Published:** 2023-12-07

**Authors:** Rafał Nowaczyński, Piotr Paszke, Andrea Csaki, Jarosław Mazuryk, Krzysztof Rożniatowski, Piotr Piotrowski, Dorota Anna Pawlak

**Affiliations:** † Faculty of Materials Science and Engineering, Warsaw University of Technology, Woloska 141, 02-507 Warsaw, Poland; ‡ Department of Chemistry, University of Warsaw, Pasteura 1, 02-093 Warsaw, Poland; § ENSEMBLE3 Centre of Excellence, Wolczynska 133, 01-919 Warsaw, Poland; ∥ Leibniz Institute of Photonic Technology, Albert-Einstein-Str. 9, 07745 Jena, Germany; ⊥ Department of Electrode Processes, Institute of Physical Chemistry Polish Academy of Sciences, Marcina Kasprzaka 44/52, 01-224 Warsaw, Poland; # Bio & Soft Matter Group, Institute of Condensed Matter and Nanosciences, Université catholique de Louvain, 1 Place Louis Pasteur, 1348 Louvain-la-Neuve, Belgium; ∇ Łukasiewicz Research Network - Institute of Microelectronics and Photonics, Wolczynska 133, 01-919 Warsaw, Poland

## Abstract

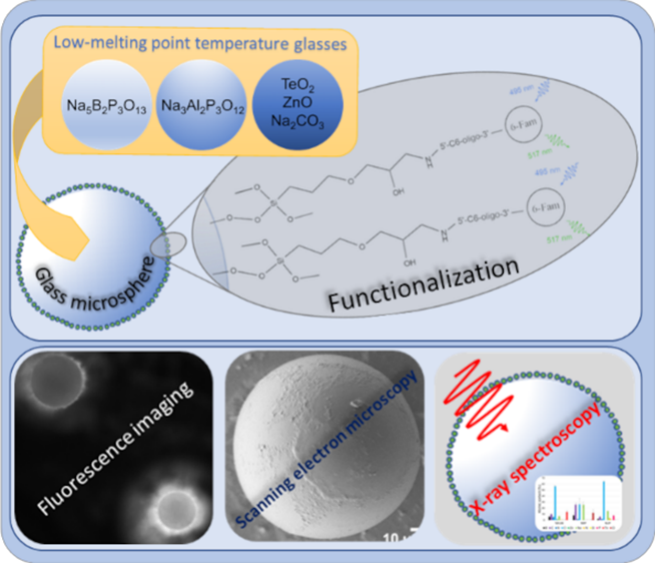

Active whispering gallery mode resonators made as spherical microspheres doped with quantum dots or rare earth ions achieve high quality factors and are excellent candidates for biosensors capable of detecting biomolecules at low concentrations. However, to produce quantum dot-doped microspheres, new low melting temperature glasses are sought, which require surface functionalization and antibody immobilization for biosensor development. Here, we demonstrate the successful functionalization of three low melting point glasses and microspheres made of them. The glasses were made from sodium borophosphate, sodium aluminophosphate, and tellurite, and then, they were functionalized using (3-glycidyloxypropyl)trimethoxysilane in ethanol- and toluene-based protocols. Proper silanization was confirmed by energy-dispersive X-ray spectroscopy and fluorescence microscopy of an amino-modified luminescent oligonucleotide probe. Fluorescence imaging showed successful silanization for all tested samples and no degradation for aluminophosphate and tellurite glasses. The strongest signal was registered for tellurite glass samples functionalized using the toluene-based silanization protocol. This conclusion implies that this functionalization method is the most efficient and is highly recommended for future antibody immobilization and biosensing application.

## 1 Introduction

Whispering gallery mode (WGM) microresonators (MRs) are optical devices capable of detecting very subtle changes in their immediate surroundings, making them excellent candidates for highly selective and sensitive sensing devices. They can be fabricated in different shapes, such as rings, discs, or in toroidal form, from glass or crystalline materials; but, the highest Q factors are provided by spherical, glass-based microresonators^1^ that can be easily manufactured by using either free-fall furnace or fiber-melting techniques.^2,3^ WGM MRs can be used for detecting biological agents, such as viruses or cancer biomarkers (e.g., exosomes and miRNA) in low concentrations^4−7^ down to single molecules, provided a proper functionalization of the microresonator surface with specific antibodies. In the case of silicate glasses abundant with −OH groups, it is possible to modify their surfaces with silanes, such as (3-glycidyloxypropyl)trimethoxysilane (GOPS).^8^ During this silanization, the silane is hydrolyzed and, as a reactive silanol, attaches to the substrate, thus creating an epoxy-group-displaying silane monolayer. The epoxy group is prone to interact and bind with functional groups of thiol-, amine-, and hydroxyl-containing ligands, thus allowing immobilizing amine-rich antibodies on the glass surface.^9^

Noticeable improvement of WGM-based sensor sensitivity can be achieved by embedding optically active elements on the MR surface.^10−13^ WGM MRs are usually doped with a gain medium to constitute microlasers that are typically exploited in barcode-type cell tagging,^14^ ultrasensitive label-free biodetection, and monitoring of molecular interactions.^13,15−18^ Nevertheless, most commonly, silica MRs are used in biosensing, relying on the WGM phenomenon. As an easily accessible and cheap core material, silica-based WGM MRs can be easily fabricated by melting a tip of an optical fiber^19−21^ or via photolithography methods.^22^ Although silanization and biofunctionalization of these types of resonators are well-known procedures,^23^ the high melting point of the silica appears troublesome as it disables direct, volumetrical, and efficient doping of the core silica with optically active, high temperature-sensitive nanoparticles. The most renowned procedure for volumetric MR doping with active nanoparticles is the use of rare earth ions as dopants.^24^ However, in recent years, increasing interest in incorporating other luminescent nanoparticles such as quantum dots in WGM lasers as an active medium is observed.^18^ Excluding rare earth ions as a candidate for dopants, silica-based WGM lasers are mainly fabricated by covalent attaching fluorescent molecules or nanoparticles onto the MR surface.^25−27^ On the other hand, coating the MR with luminescent molecules or nanoparticles limits effective attachment of the biological receptor element (BRE, e.g. antibodies), thus hampering biodetection. One of the methods of efficient volumetric WGM MR doping is the use of polymer-based WGM, e.g., PMMA, PDMS, and polystyrene.^28−33^ However, lasing in doped polymer MRs is unstable because of the photobleaching effect.^34^ Other examples of gain media for WGM lasers are perovskites^35,36^ and semiconductor oxides.^37,38^ Unfortunately, these inorganic compounds are not convenient for biodetection because the attachment of the BRE to so-doped MR surfaces is challenging.

Therefore, for creating active, stable spherical resonators for future biosensing, glasses with lower melting points can be used. In this regard, phosphate and tellurite glasses are excellent candidates for fabricating WGM MRs.^39^ Low melting points of these glasses enable easy doping with various kinds of optically active elements by using a nanoparticle direct doping method (NPDD).^40−42^

**Figure 1:**
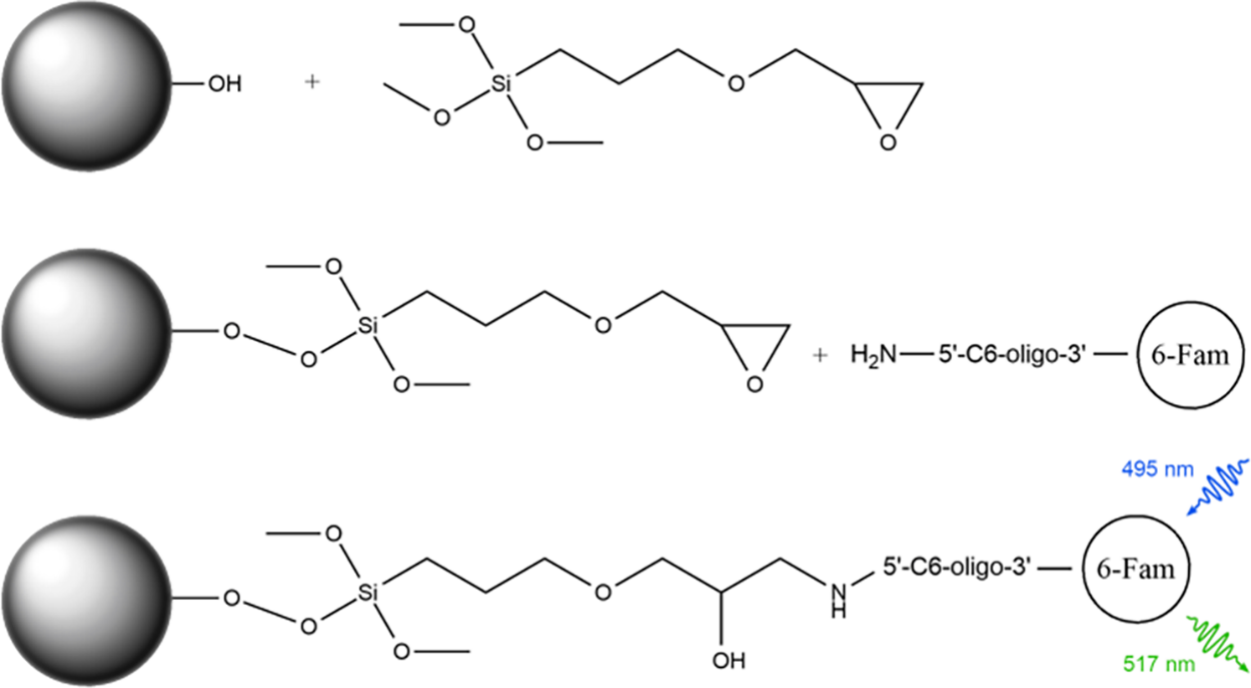
GOPS silanization, oligonucleotide bioconjugation, and detection of a fluorescent 6-Fam die. In the first step, epoxysilane attaches itself to hydroxyl groups, creating a monolayer on the glass surface. In the next step, amino-modified fluorescent oligonucleotide attaches to the epoxysilane monolayer through secondary amine formation. The presence of the oligonucleotide on the glass surface is determined using fluorescence microscopy.

Here, we demonstrate the use and biofunctionalization of low melting phosphate and tellurite glasses for future WGM biosensing. The low melting point sodium borophosphate (Na_5_B_2_P_3_O_13_, NBP, *m*_p_ = 750 °C),^43^ sodium aluminophosphate (Na_3_Al_2_P_3_O_12_, NAP, *m*_p_ = 950 °C),^44^ and tellurite (80% mol TeO_2_, 10% mol ZnO, 10% mol Na_2_CO_3_, TZN-80, *m*_p_ = 450 °C)^45^ glasses were produced using an NPDD method and then used for fabricating MRs. The glasses and MRs were successfully silanized with GOPS, which was confirmed by scanning electron microscopy with energy-dispersive X-ray photoelectron spectroscopy (SEM-EDX) and fluorescence microscopy of a bioluminescent marker, bioconjugated to the MR surfaces using a C6 aminolinker of a 6-Fam-labeled oligonucleotide (Figure 1).

## 2 Results and Discussion

### 2.1 Silanization of Glass Powder

For silanization, NAP, NBP, and TZN glasses were separately ground to a powder and divided into three samples: the first one was GOPS-silanized in EtOH using an ethanol (EtOH) protocol,^46^ the second GOPS-silanized in toluene,^47^ and the third one with no silanization. All of these glasses were then subjected to bioconjugation with a 6-Fam-labeled oligonucleotide in an aqueous solution.

**Figure 2:**
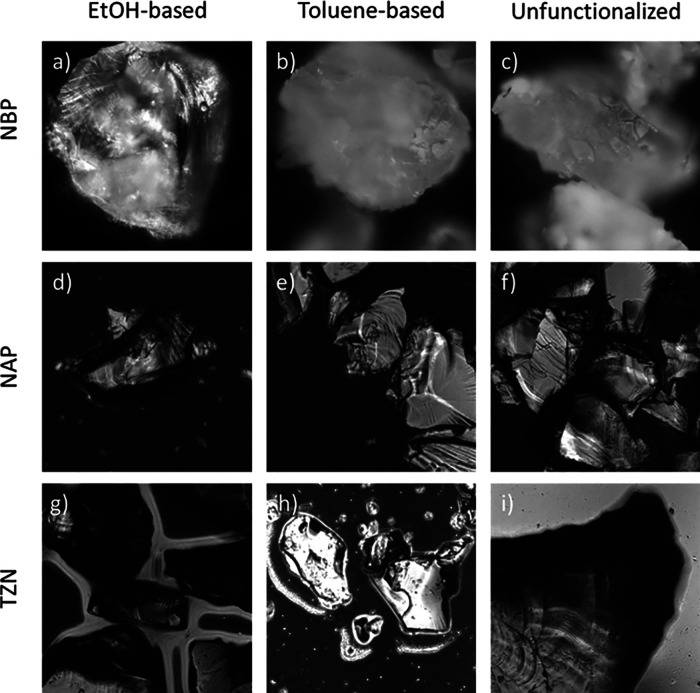
Dark-field images of functionalized glasses: (a–c) NBP glass; (d–f) NAP glass; and (g–i) TZN glass. Images (a), (d), and (g) show the glasses functionalized using the EtOH-based protocol; images (b), (e), and (h) show the glasses functionalized using the toluene-based protocol; images (c), (f), and (i) show the glasses without GOPS silanization but after bioconjugation with the 6-Fam-labeled oligonucleotide.

**Figure 3:**
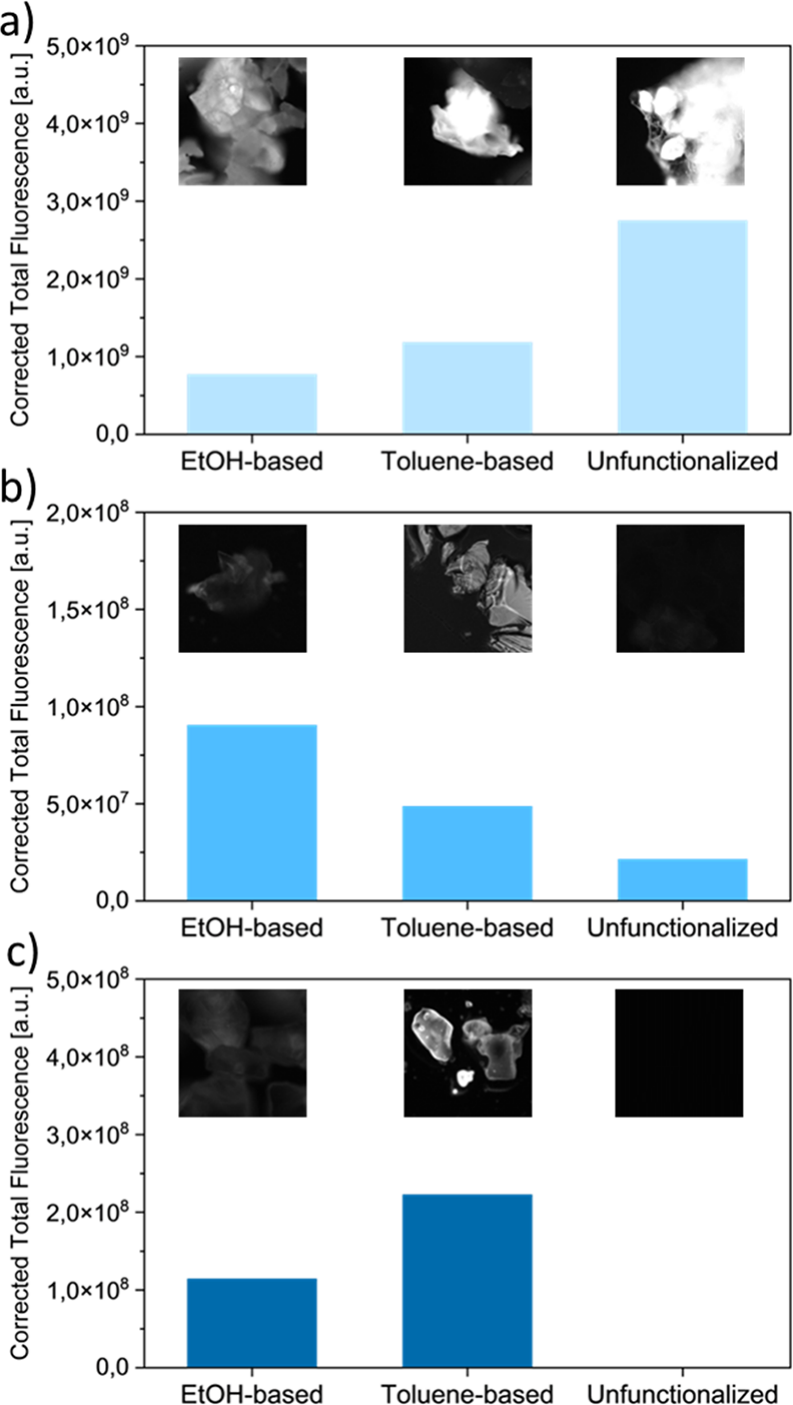
Corrected total fluorescence plots of glass powders: (a) NBP, (b) NAP, and (c) TZN. These glasses were either functionalized with the EtOH protocol and bioconjugated; functionalized with the toluene protocol and bioconjugated; or bioconjugated without functionalization. Inset pictures: fluorescence images of respective glass particles.

The first glass tested was NBP (sodium borophosphate glass, Na_5_B_2_P_3_O_13_, *m*_p_ = 750 °C).^43^ The dark field optical microscopy of NBP, silanized in EtOH and subsequently bioconjugated with a fluorescent oligonucleotide, showed NBP glass powder grains with retained initial shape with well-defined, sharp edges and fracture planes (Figure 2a). In fluorescence mode, a visible glow of glass powder was detected, which confirms the presence of the oligonucleotide on the surface (Figure 3a, left inset). In contrast, when a toluene-based functionalization solution and bioconjugation were applied to NBP, the aspect of the glass grains changed, and sharp edges and clean fracture planes were no longer visible (Figure 2b). This observation indicates that the conditions of NBP functionalization or reaction led NBP degradation. Nonetheless, despite this degradation, the bright fluorescence of the NBP-oligonucleotide material (Figure 3a, middle inset) confirms successful bioconjugation. Indeed, the NBP glass powder suspended in an aqueous 6-Fam-labeled oligonucleotide solution resulted in similar glass powder grain degradation to that in toluene (Figure 2c) due to the hygroscopic properties of NBP. Fluorescence imaging (Figure 3a, right inset) and quantitative fluorescence analysis were performed by measuring the corrected total fluorescence (CTF, Figure 3a). However, the fluorescence images display a significant glow of the material, whereas CTF analysis of the glass grains revealed the strongest signal for the unmodified sample (Figure 3a, right inset) when compared to NBP-oligonucleotide bioconjugates (Figure 3a, left and middle insets). We explain this fluorescence by the fact that the oligonucleotide should not be able to bond chemically to the glass surface without epoxy groups, but it can still be present due to nonspecific physical bonding, especially when the active surface area is greatly developed as a result of the glass suspension in water. The intense fluorescence of the oligonucleotide results from the entrapment of the oligonucleotide in spaces of the degraded NBP, which disallows its easy separation from the glass via washing. Because no degradation was observed in the case of the in-EtOH-prepared NBP, it may be assumed that a silane monolayer protected the glass surface from water during bioconjugation. In contrast, this effect was not observed for in-toluene-prepared NBP because toluene itself may cause NBP glass degradation.

In the case of NAP (sodium aluminophosphate glass, Na_3_Al_2_P_3_O_12_, *m*_p_ = 950 °C),^44^ dark field and fluorescence microscopy revealed that both silanized and conjugated NAP samples exhibited a nondegraded structure with well-defined sharp edges and fracture planes of glass grains (Figure 2d–f, Figure3b). Based on the fluorescence imaging and the CTF analysis of the oligonucleotide, we concluded the highest intensity of the NAP-oligoconjugate fabricated in EtOH (Figure 3b). Without silanization, only slightly visible fluorescence was detected, presumably resulted from a weak, nonspecific physical bonding, including adsorption or physisorption. Moreover, nonfunctionalized NAP glass did not degrade when exposed to an aqueous solution of the oligonucleotide.

Finally, surfaces of TZN-80 glass grains (80% mol TeO_2_, 10% mol ZnO, 10% mol Na_2_CO_3_, TZN-80, *m*_p_ = 450 °C)^45^ were functionalized by silanization and bioconjugation. Similar to NAP glass, both EtOH- and toluene-based functionalization of the TZN-80 surfaces in an efficient and degradation-free manner were proven by dark field and fluorescence microscopy (Figure 2g–i, Figure 3c). Indeed, the TZN-80-oligonucleotide conjugate obtained in the toluene-based procedure exhibited the highest fluorescence intensity among all glasses tested in the present study. Moreover, nonfunctionalized TZN-80 did not display any fluorescence. These conclusions indicate TZN-80 as the most suitable low melting point glass for biofunctionalization and future optical/WGM biosensing applications.

### 2.2 Silanization of Glass Microspheres

After optimization of silanizing surfaces of NBP, NAP, and TZN-80 glasses, these glasses were used for fabricating spherical MRs, using a free-fall furnace^2^ followed by toluene-based silanization with GOPS and subsequent bioconjugation with a C6 aminolinker of the 6-Fam-labeled oligonucleotide. Toluene was chosen for MR silanization because of its superior performance revealed in the case of the TZN-80 glass.

**Figure 4:**
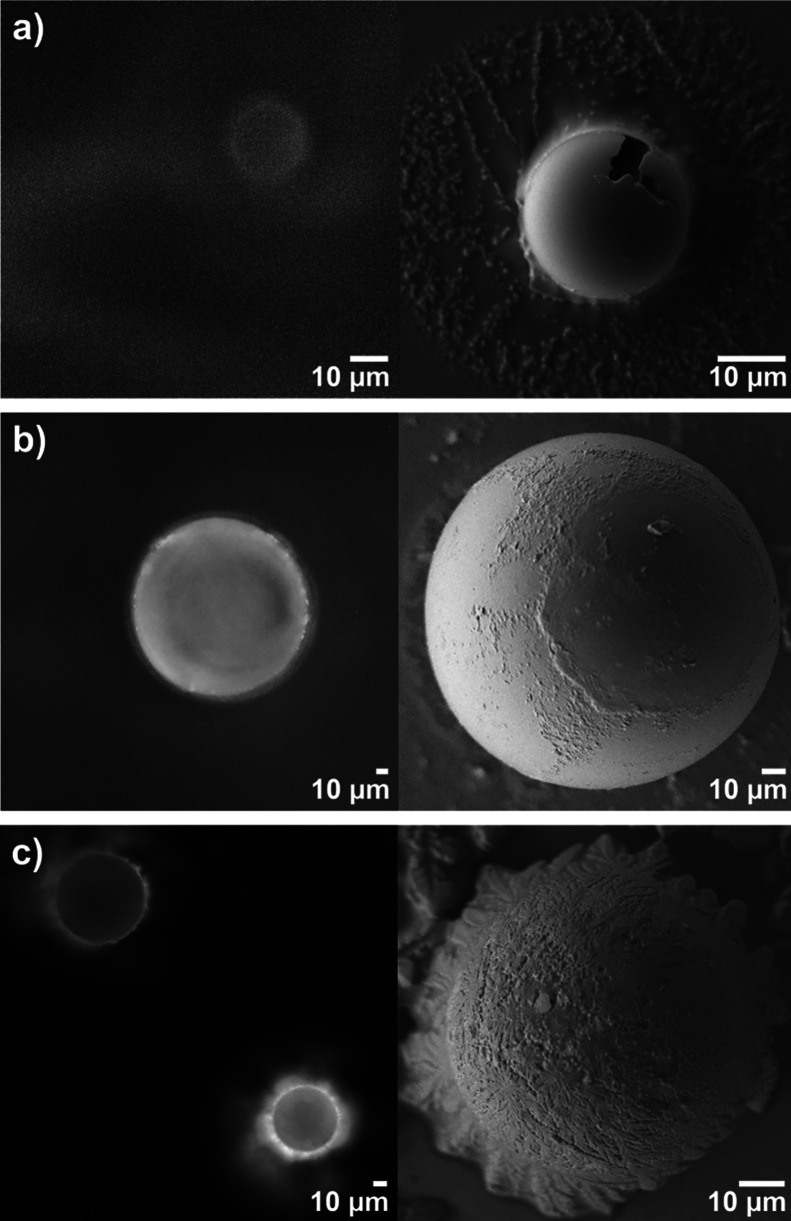
Glass microspheres after silanization and bioconjugation: (a) NBP, (b) NAP, and (c) TZN-80. Left: fluorescence optical microscopy; right: scanning electron microscopy. Results demonstrate successful silanization of NAP and TZN-80 glass microspheres without visible degradation and the degradation of NBP glass microspheres.

Similar to measurements of patterned glass powders, the silanization of NBP glass was successful but the MR displayed a poor chemical stability manifested by MR surface degradation (Figure 4a). In contrast, NAP MRs were successfully silanized and biofunctionalized with a fluorescent oligonucleotide. Good quality and no degradation of the NAP MRs were demonstrated by SEM imaging (Figure 4b). Likewise, SEM and fluorescence imaging of TZN-80 MRs confirmed the successful silanization and bioconjugation without surface degradation. (Figure 4c).

**Figure 5:**
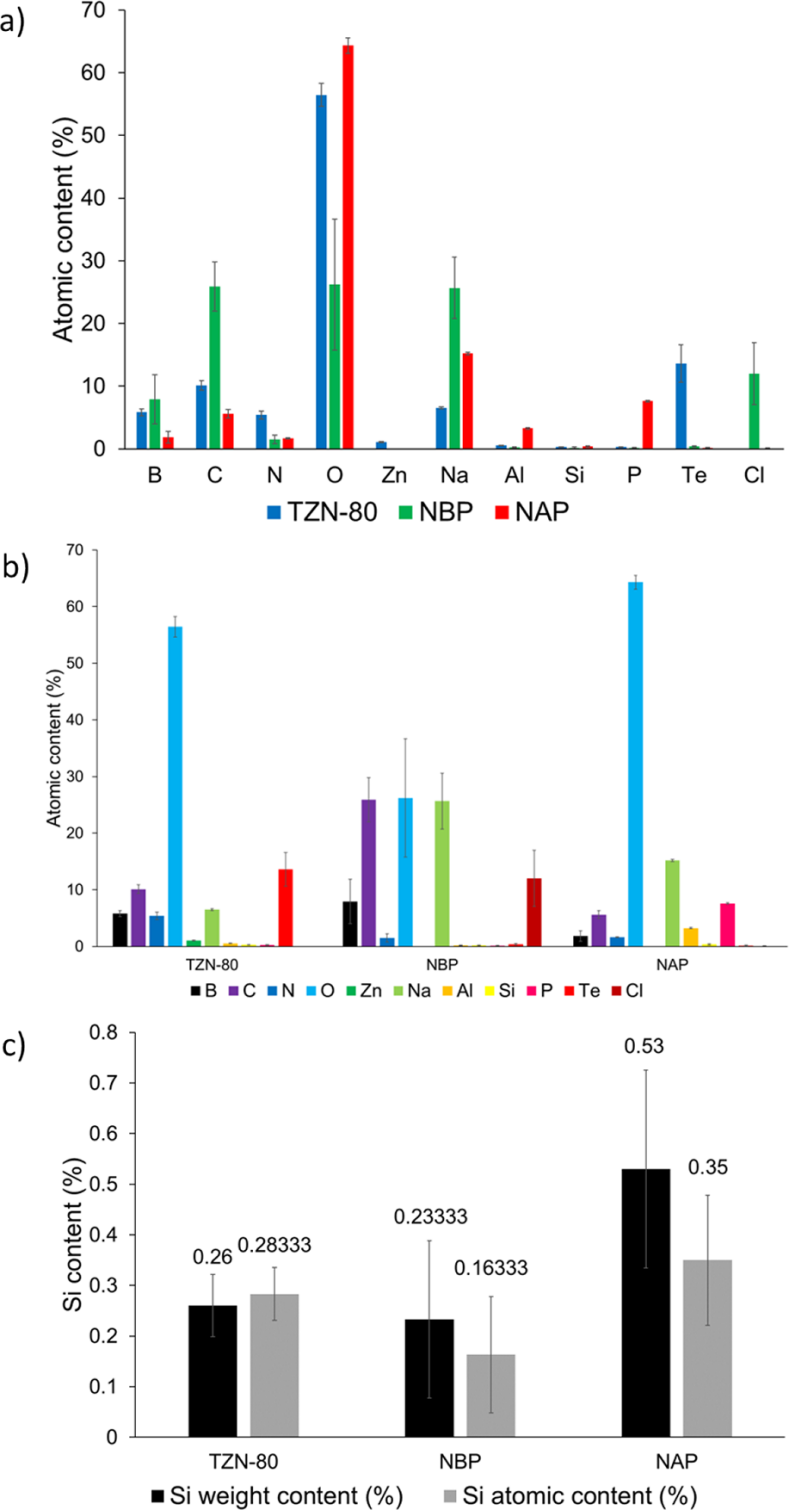
Elemental distribution and elemental atomic contents in TZN-80, NBP, and NAP microresonator glasses, functionalized with GOPS-oligonucleotide-6-Fam, with regard to (a) the element, (b) the glass, and (c) the Si content.

The efficiencies of GOPS-silaned TZN-80, NBP, and NAP MRs were analyzed by EDX spectroscopy. This approach was applied to measure the silicon content in the silanized samples since GOPS is the only source of silicon in the samples of interest. Quantitative analysis of the EDX spectra is presented in Table 1 and Figure 5. Figure 5a,b presents the percentage atomic contents for the three types of glasses. As shown, the analysis confirmed the elemental distribution in the glass MRs, expressed by their structural formulas, as follows: TZN-80 (TeO_2_, ZnO_2_, and Na_2_CO_3_), NBP (Na_5_B_2_P_3_O_13_), and NAP (Na_3_Al_2_P_3_O_12_). The silicon content in the glasses, presented in Figure 5c, clearly demonstrates efficient silanization with GOPS. Since the silanization was optimized to obtain a GOPS monolayer on the MR surface, the weight and atomic Si contents are relatively low in comparison to the major elements of the glasses. Finally, the EDX analysis indirectly confirms bioconjugation of oligonucleotide-6-Fam to GOPS-functionalized TZN-80. As shown in Table 1, atomic contents of P (0.26 ± 0.064) and Si (0.283 ± 0.052) are statistically comparable, which suggests efficient GOPS-oligonucleotide-6-Fam bioconjugation in a 1:1 molar ratio. This cannot be concluded for NBP and NAP glasses as P is intrinsically present in these glasses.

**Table 1:** EDX Analysis of the NAP, NBP, and TZN-80 Glass Microspheres Functionalized with GOPS and Oligonucleotide-6-Fam^a^

	**TZN-80**				**NBP**				**NAP**			
	**weight content (%)**		**atomic content (%)**		**weight content (%)**		**atomic content (%)**		**weight content (%)**		**atomic content (%)**	
element	**mean**	**SD**	**mean**	**SD**	**mean**	**SD**	**mean**	**SD**	**mean**	**SD**	**mean**	**SD**
B	2.023	0.309	5.803	0.527	4.350	1.994	7.930	3.925	1.070	0.562	1.817	0.939
C	3.940	0.743	10.123	0.785	16.543	3.854	25.883	3.905	3.663	0.488	5.613	0.688
N	2.457	0.584	5.383	0.658	1.090	0.467	1.507	0.710	1.260	0.041	1.657	0.066
O	29.150	4.344	56.430	1.824	22.717	10.940	26.197	10.453	55.857	0.715	64.307	1.213
Zn	2.203	0.100	1.057	0.075	0.000	0.000	0.000	0.000	0.000	0.000	0.000	0.000
Na	4.803	0.460	6.507	0.164	30.590	3.867	25.670	4.915	18.960	0.282	15.193	0.199
Al	0.473	0.071	0.540	0.016	0.253	0.108	0.183	0.084	4.757	0.124	3.247	0.109
Si	0.260	0.062	0.283	0.052	0.233	0.155	0.163	0.115	0.530	0.195	0.350	0.128
P	0.257	0.045	0.260	0.064	0.177	0.100	0.113	0.066	12.773	0.232	7.597	0.144
Te	54.437	6.539	13.607	2.987	2.397	0.698	0.363	0.123	0.977	0.683	0.143	0.101
Cl	0.000	0.000	0.000	0.000	21.650	8.008	11.987	4.956	0.150	0.115	0.077	0.061

a Each sample was measured three times. Results are presented as mean ± SD.

**Figure 6:**
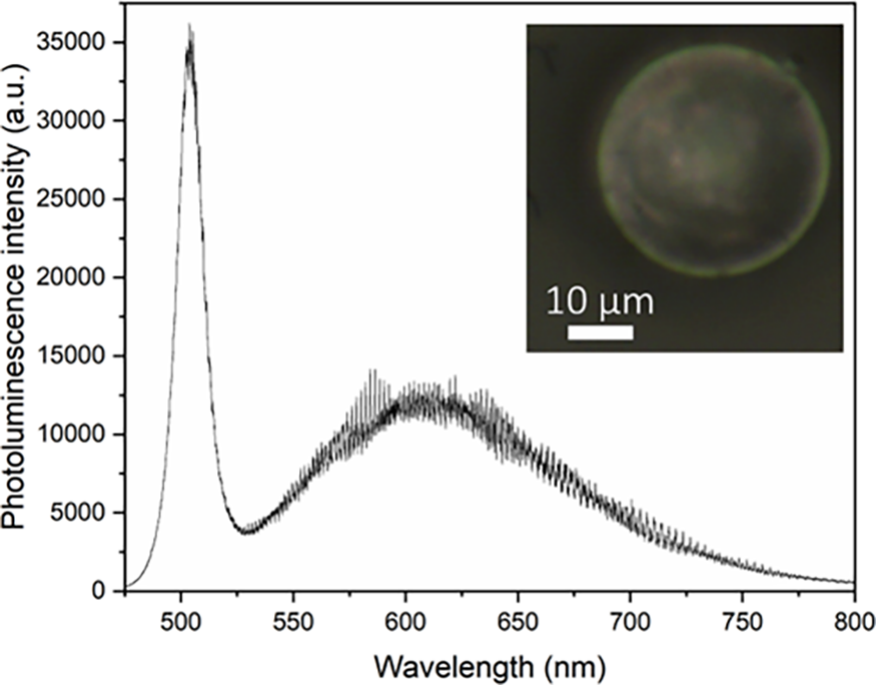
WGM resonance pattern on the emission of an NBP glass microsphere doped with 0.4 wt % silver nanoparticles and 0.3 wt % CdTe quantum dots. Inset: image of the measured microsphere.

Raw and doped MRs based on low melting point glasses, such as NAP, NBP, and TZN-80, are expected to exhibit a WGM resonance. For instance, recently, using NBP glass as a matrix, we obtained WGM-displaying microspheres based on doped NBP MRs with quantum dots and plasmonic nanoparticles. [Piotr Paszke et al., “Plexcitonic spherical glass whispering gallery mode microresonators exhibiting amplified narrowband emission”, in preparation]. A WGM pattern of these doped ∼36-μm diameter NBP MRs is presented on Figure 6. The image shows excitonic and defect emission of quantum dots with maximum wavelengths of 504 and 617 nm, respectively. The excitation wavelength was 473 nm. A characteristic WGM pattern could be seen throughout the entire range of the emission spectrum. However, it must be highlighted that our present study does not present Q factors of the fabricated MRs. Although the Q factor measurements were not a major goal of this study, they will be carried out in the near future as the Q factors represent essential properties of all WGM MRs. Future measurements are envisioned to demonstrate WGM resonances of biofunctionalized or doped NAP and TZN-80 MRs. Also, much effort will be done in demonstrating WGM-mediated biosensing properties of the glasses using real-world samples.

## 3 Conclusions

Low melting point NBP, NAP, and TZN-80 glasses were successfully silanized in powdered form using GOPS in toluene and EtOH solvents. Based on this optimization, respective MRs were fabricated and silanized using a toluene-based procedure, demonstrated as effective for NAP and TZN-80 glasses. EtOH-based silanization, while being less effective for TZN-80, could potentially be used for NBP glass due to its degradation-preventing properties. Silanization efficiencies were confirmed using EDX spectroscopy, which confirmed the Si content in the composites, and by fluorescence microscopy, which visualized the successful bioconjugation of a fluorescent amino-modified oligonucleotide to silanized surfaces of glass powders and MRs. These oligonucleotides attach to the surface of silane-modified glass by forming a chemical bond between the GOPS epoxy group and C6 aminolinker at the 5′ end of the nucleotide, which emulates the amines present in antibodies.

This result demonstrates that the silanization and bioconjugation may be useful to immobilize antibodies containing primary amines on the surface of easily fabricated phosphate and tellurite glass-based microspheres, especially NAP and TZN-80. The low melting points of these glasses enable easy doping of these materials with optically active nanoparticles using physical methods.^40−42^ As such, they can act as active lasers for sensitive and selective biosensors in WGM-based sensing devices.^18^ These kinds of active WGM resonators are already used in (i) barcode-type cell tagging and tracking^48^ and (ii) DNA,^49,50^ single nanoparticle, and virus detection.^16^ Due to its susceptibility to degradation in aqueous and toluene-based solutions, NBP glass should be avoided in these applications.

## 4 Materials and Methods

### 4.1 Silanization with GOPS Ethanol Protocol

A 40 mL portion of silanization solution was prepared using 37.3 mL of 99.8% ethanol, 1.9 mL of Milli-Q water, and 0.8 mL of (3-glycidyloxypropyl)trimethoxysilane. Glass powder or microspheres were put in the solution and incubated with mixing at 37 °C overnight. After the incubation, the glass was washed twice in EtOH, once in Milli-Q water, and then dried.

### 4.2 Silanization with GOPS Toluene Protocol

A 40 mL portion of silanization solution was prepared using 39.9 mL of extra dry toluene and 0.1 mL of (3-glycidyloxypropyl)trimethoxysilane mixed under an argon atmosphere. Glass powder or microspheres were put in the solution and incubated with mixing at 70 °C overnight. After the incubation, the glass was washed twice in toluene, twice in EtOH, and once in Milli-Q water and then dried.

### 4.3 Bioconjugation

For the bioconjugation procedure, a 10 μM solution of amino-modified nucleotides (H_2_N-5′-C6-tcg ttt tat cgg gcg gaa tg-3′-6-Fam, 00071721-6 from biomers.net GmbH) in a 2× saline sodium citrate (SSC) buffer was prepared. Silanized glass powder/microspheres were placed in the solution at 4 °C overnight. After bioconjugation, the glass was washed once in Milli-Q water, dried, and characterized using fluorescence microscopy and scanning electron microscopy. From the fluorescence images, corrected total fluorescence was calculated in a fashion similar to the corrected total cell fluorescence method used in biological studies using the following formula: CTF = integrated density(area of selected glass grain × mean fluorescence of background readings).

### 4.4 Energy-Dispersive X-ray Spectroscopy

Elemental analysis of NAP, NBP, and TZN-80 glasses, functionalized with GOPS silane and an oligonucleotide, was performed using EDX spectroscopy coupled with scanning electron microscopy (SEM). The measurements were conducted using an FEI Nova NanoSEM 450 equipped with an EDX spectrometer. For the measurements, the glass samples were drop-cast on gold plates and dried overnight. Each glass sample was measured three times. Results were plotted and presented as a mean of weight and atomic contents (wt % and at %, respectively) ± standard deviation (SD). Raw EDX spectra are available in the Supporting Information.

## Supporting Information Available

Raw EDX spectra of silicon plate and TZN-80, NBP, and NAP glasses (PDF)

## List of abbreviation

BRE: biological receptor element
CTF: corrected total fluorescence
GOPS: (3-glycidyloxypropyl)trimethoxysilane
MR: microresonator
NAP: sodium aluminophosphate glass
NBP: sodium borophosphate glass
NPDD: nanoparticle direct doping method
SEM-EDX: scanning electron microscopy with energy-dispersive X-ray photoelectron spectroscopy
TZN: tellurite glass
WGM: whispering gallery mode
