# Supplementary material for: Functionalization of Phosphate and Tellurite Glasses and Spherical Whispering Gallery Mode Microresonators
Source: ACS Omega. 2023 Dec 7;8(50):48159–65. doi: 10.1021/acsomega.3c07075 (PMC10734010; doi:10.1021/acsomega.3c07075)
Supplement: Supplementary file 1 — ao3c07075_si_001.pdf [file ao3c07075_si_001.pdf]

Supporting Information

Functionalization of phosphate and tellurite glasses and spherical WGM microresonators

Rafał Nowaczyński[1,2*], Piotr Paszke[2,3*], Andrea Csaki[4], Jarosław Mazuryk[5,6], Krzysztof Rożniatowski[1], Piotr Piotrowski[2,3] and Dorota Anna Pawlak [2,3,7*]

[1] *Faculty of Materials Science and Engineering, Warsaw University of Technology, Woloska 141, 02-507 Warsaw, Poland.*

[2] *Department of Chemistry, University of Warsaw, Pasteura 1, 02-093 Warsaw, Poland.*

[3] *ENSEMBLE[3] Centre of Excellence, Wolczynska 133, 01-919 Warsaw, Poland.*

[4] *Leibniz Institute of Photonic Technology, Albert-Einstein-Str. 9, 07745, Jena, Germany.*

[5] *Department of Electrode Processes, Institute of Physical Chemistry Polish Academy of Sciences, Marcina Kasprzaka 44/52, 01-224 Warsaw, Poland*

[6] *Bio & Soft Matter Group, Institute of Condensed Matter and Nanosciences, Université catholique de Louvain, 1 Place Louis Pasteur, 1348 Louvain-la-Neuve, Belgium*

[7] *Łukasiewicz Research Network - Institute of Microelectronics and Photonics, Wolczynska 133, 01-919 Warsaw, Poland.*

Raw EDX spectra

<table>
<tr><th>TZN-80 (glass #1)</th><th>TZN-80 (glass #2)</th></tr>
<tr><td>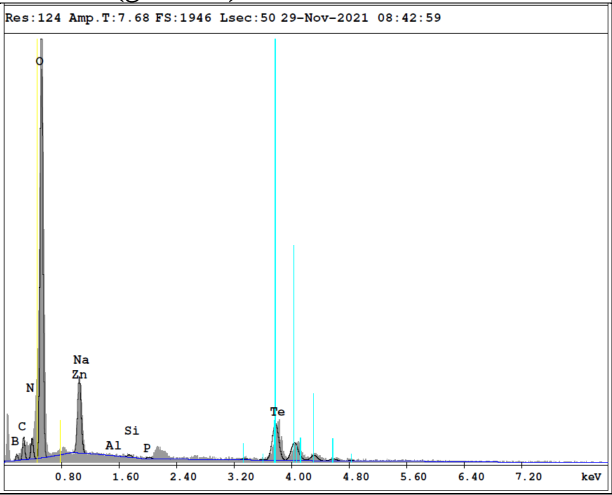
</td><td>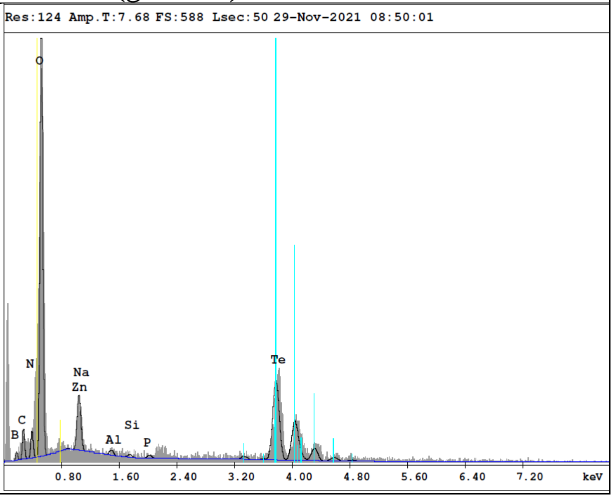
</td></tr>
</table>

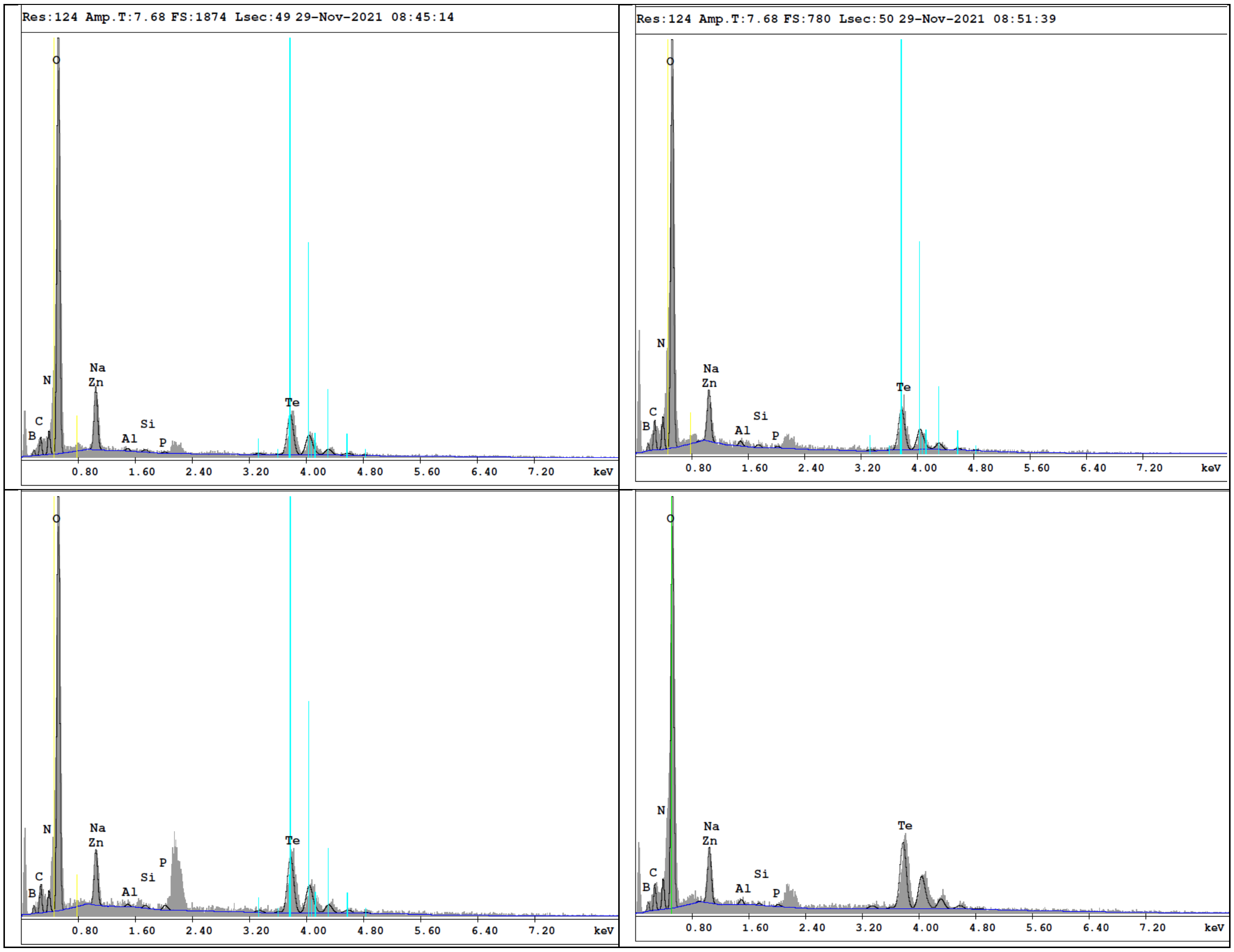

Figure S1 Raw EDX spectra of TZN-80 glasses.

NBP | NAP (glass #4)

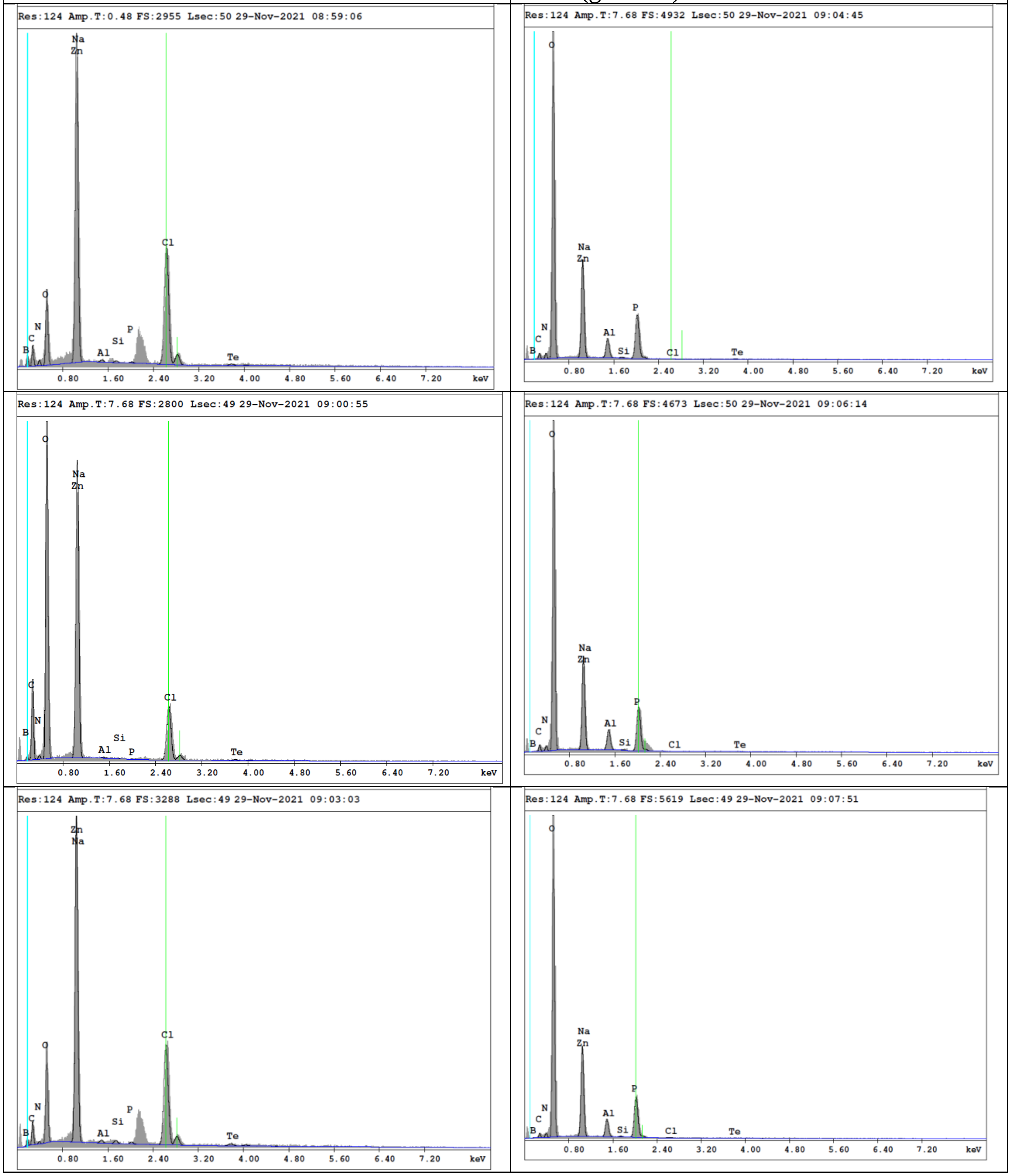

Figure S2 Raw EDX spectra of NBP and NAP glasses.

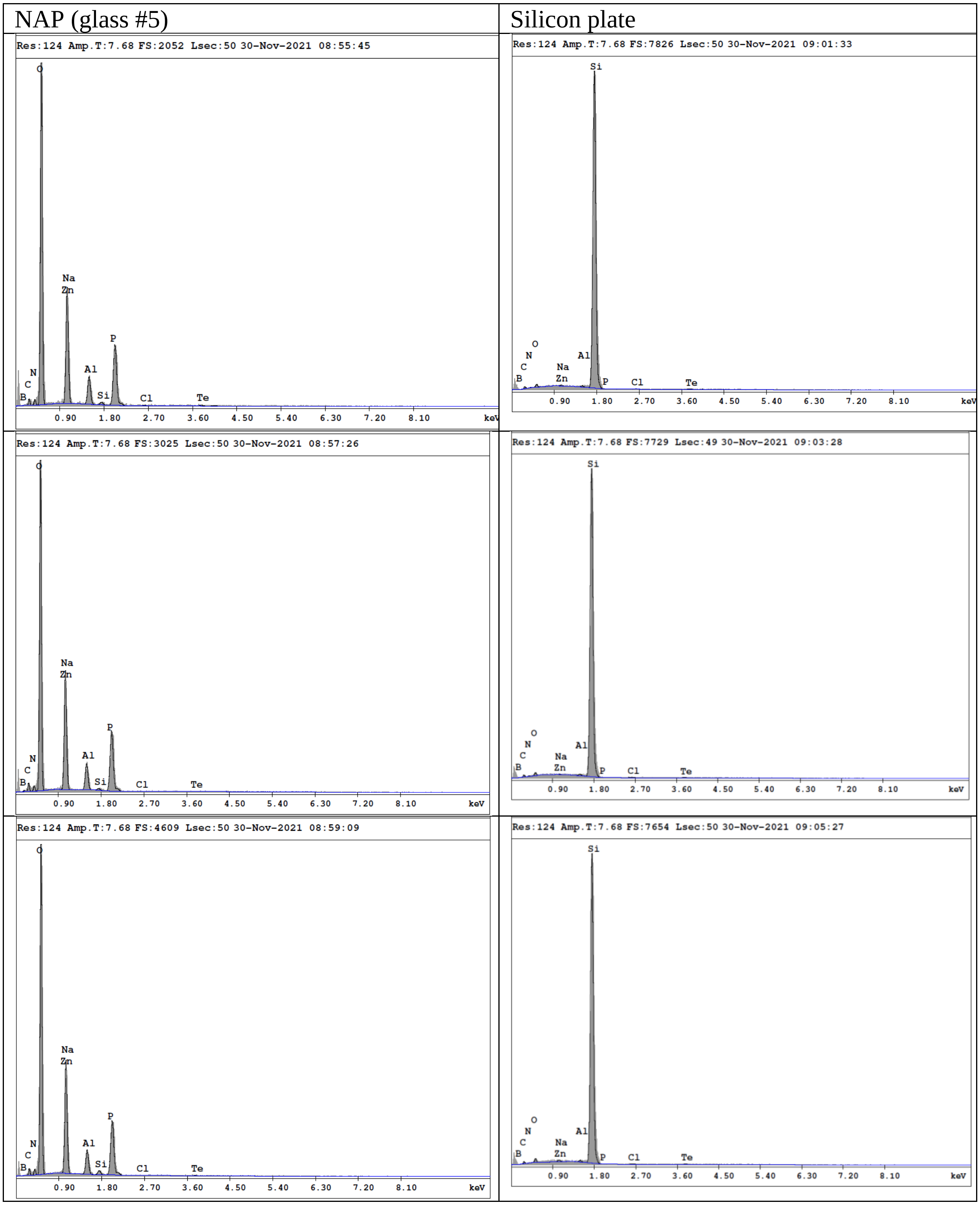

Figure S3 Raw EDX spectra of NAP glass and silicon plate.
